# Creativity in Motion: Examining the Creative Potential System and Enriched Movement Activities as a Way to Ignite It

**DOI:** 10.3389/fpsyg.2021.690710

**Published:** 2021-09-30

**Authors:** Veronique Richard, Darren Holder, John Cairney

**Affiliations:** ^1^National Circus School, Center for Circus Arts Research, Innovation and Knowledge, Montreal, QC, Canada; ^2^Coaching Better, Brisbane, QLD, Australia; ^3^School of Human Movement and Nutrition Sciences, University of Queensland, St Lucia, QLD, Australia

**Keywords:** creativity, movement intervention, non-linear pedagogy, improvisation, ecological dynamics, physical literacy, embodiment

## Abstract

In a global and highly competitive world, the importance of creativity is increasing as it supports adaptability, health, and actualization. Yet, because most research focuses on what it takes to produce creative artifacts, interventions supporting growth in creative potential remains underexplored. To address this limitation, the first goal of this paper is to review the creativity science literature to identify the elements that underpin the realization of an individual’s creative potential. The summary of the literature is presented using a framework which highlights the interactions between environmental elements (i.e., cultural values, social interactions, and material world) and actors’ elements (i.e., affective attributes and states, cognitive skills, and physical expression). Using a systemic perspective, the framework illustrates ‘what’ creativity enhancement interventions should aim for, to facilitate the emergence of creative actions. Given the current lack of holistic, embodied, and interactive evidence-based interventions to nurture the creative potential elements identified, the second part of this review builds on movement sciences literature and physical literacy conceptualization to suggest that enriched movement activities are promising avenues to explore. Specifically, following non-linear pedagogy approaches, an intervention called movement improvisation is introduced. Ecological dynamics principles are used to explain how improvising with movement in a risk-friendly environment can lead to cognitive, affective, social, and cultural repertoire expansion. To interrogate this argument further, the review concludes with possible solutions to withstand research challenges and raises future study questions. Overall, combining creativity and movement sciences in this review demonstrates the potential for well-designed movement interventions to ignite creative potential for individuals and overcome the tendency to remain anchored in a state of inertia.

## Introduction

We live in a world in constant motion where continuous changes are taking place at all levels (Glăveanu, 2010a). To adapt to this ever-changing world and solve the problems that changes might engender from daily hassles to wicked societal issues, creativity has been identified by organizations around the world (e.g., The Partnership for 21st Century Learning, Bloom’s Taxonomy) as one of the differentiating skills (Hennessey and Amabile, 2010; Barbot et al., 2015; Glăveanu, 2018; Malinin, 2019). In addition to its association with adaptability, psychologists consider creativity as being instrumental for human actualization, self-expression, and health (Runco, 2004; Glăveanu, 2010a). Consequently, a growing number of scholars are studying this skill to better capture its antecedents, components, and mechanisms (Hennessey and Amabile, 2010; Choi et al., 2020). As a result of this research endeavor, creativity developed from being defined as the generation of ideas, insights, or solutions that are new and meant to be useful (Sternberg and Lubart, 1999, p.411) to a much more complex and integrated definition. According to Glăveanu’s (2013) *Five A’s Framework*, “creativity is concerned with the *action* of an *actor* or group of *actors*, in its constant interaction with multiple *audiences* and the *affordances* of the material world, leading to the generation of new and useful *artifacts*” (p.76). In line with this definition, creativity research has also shifted from a computational (top-down) cognitive model where creativity happens in the mind first and is then transformed in behaviors to an embodied conceptualization where “the mind is not solely located in the brain but also involves the body and the body’s situation in the environment” (Malinin, 2019, p.2).

It is this holistic, embodied, and interactive conceptualization of creativity that raises some challenges when it comes to building effective interventions designed to nurture *creative potential* (Vaughan et al., 2019). Creative potential “may be awakened through favorable experiences, training, environments, or stifled by negative versions of these same elements” (Corazza and Glăveanu, 2020, p.2). In fact, scientists have not yet clearly identified which pedagogical practices lead to creative potential enhancement (Barbot et al., 2015; Valgeirsdottir and Onarheim, 2017). Empirical evidence supporting the effectiveness of interventions targeting a holistic interplay between the external world and the internal one is lacking (Torrents et al., 2020), and few interventions are purposefully designing environmental conditions while challenging the actor’s internal stability to promote the emergence of skills leading to creative actions. Moreover, the intertwined connection between the body, the mind, and the environment has been overlooked excluding the role of the body or its physical context from most creativity enhancement interventions (Malinin, 2019).

To address these limitations, this paper critically reviews the creativity and movement sciences literature to suggest future development in the field of creativity enhancement interventions. Specifically, we aim to scientifically support the relevance of using enriched movement activities as a means to realize an individual’s creative potential. By combining existing creativity and movement theories, the goal is to provide researchers and practitioners a framework to influence the actor-environment interactions through enriched movement activities. When thoughtfully designed, these movement activities could help people explore, unfold, and better exploit their creative potential at the everyday level (see creative potential elements section next) to reach higher psychological functioning and overall wellbeing. In line with a socio-cultural manifesto written by 20 established creativity scholars, we thus view creativity enhancement interventions as a way to expand how “people relate to the world, to others, and to themselves, making them more flexible, more open to the new and, at least in principle, to differences in perspective” (Glăveanu et al., 2020, p.743).

To achieve these goals, the first section defines relevant terms and reviews mainstream creativity theories, concepts, and frameworks to connect the interacting actor and environmental elements underpinning creative potential. To build a strong argument supporting the benefits of movement interventions on these elements, the second section reviews movement sciences literature to highlight the principles underlying the design of enriched movement activities. The third section introduces *movement improvisation* to exemplify how enriched movement activities can ignite creative potential growth while the fourth section explains the mechanisms of change occurring when one engages in such activities. Finally, the fifth section raises challenges and future research directions.

## The Creative Potential Elements

The term *creative potential* is key in this review and must be carefully defined to situate and focus our perspective. Corazza and Glăveanu (2020) identified 15 different forms of creative potential issued from various philosophical and theoretical perspectives. Because creativity was defined previously using the *Five A’s Framework* (Glăveanu, 2013), thereby emphasizing a sociocultural approach to creativity, this review focuses on *embedded individual potential.* In this conceptualization, the potential for originality and effectiveness of an actor is intertwined with a sociocultural context. It is thus multifaceted and presents a unique combination of resources that interact together within the creative phenomenon (Barbot et al., 2015; Corazza and Glăveanu, 2020). When the actor and the environmental resources ‘match,’ the creative potential can be actualized increasing the likelihood of creative achievements.

According to the 4Cs framework (Kaufman and Beghetto, 2009), creative achievements can be distributed along a developmental continuum starting from mini-c (personally novel and meaningful interpretation of experiences, actions, ideas), to little-c (everyday creative achievements) and pro-c (professional creative achievements and innovations), ending (rarely) with Big-C (eminent creative contribution). While we acknowledge that pro-c and Big-C achievements require resources that are specific to a domain of expertise (Sternberg, 2012; Baer, 2015), the current review focuses on everyday level creativity (little-c) and thus targets the development of ‘general’ actor and environmental resources (also called creativity-relevant skills by Amabile and Pillemer, 2012). Little-c achievement can take the form of behavioral expression such as pretend play (especially in children), questioning, taking risk, enacting as-if, and so on (Glăveanu and Kaufman, 2019). Although little-c achievements may be optional in one’s life, these activities are associated with wellbeing and other professional benefits (Corazza, 2017). Societies should thus develop policies to “allow its members to live a life in which there is room for creative endeavors, at least at the little-c level” (Corazza and Glăveanu, 2020, p.8).

Before we explore in depth how enriched movement activities can provide opportunities for these creative endeavors, it is essential to pinpoint the key elements that constitute the *embedded individual creative potential*. Specifically, the next section identifies the environmental and actor’s elements that have been theoretically associated with enhanced creative potential and provide empirical examples highlighting their intricate interactions. We believe this step is essential to move away from the prominent ‘silo approach’ currently influencing creativity enhancement interventions. By gathering various theories, concepts, and evidence into a framework, we aim to expand the horizon of intervention and provide a holistic foundation for the design of enriched movement activities intended at fostering creative potential.

### Environmental Elements

The notion of potential is closely associated with what humans can do effectively in an environment and thus it relies heavily on both social and material elements and their relation to each other (Corazza and Glăveanu, 2020). Nevertheless, many creativity theories and frameworks emphasize the creative actor and depict the environment as an external factor that either constrains or facilitates the creative process (Glăveanu, 2010b). As such, the role of the environment is over-simplified as a conditioning factor experienced by the actor. By contrast, the *tetradic cultural framework of creativity* (Glăveanu, 2010a) moves beyond this unidirectional perspective by suggesting that “creativity is not simply ‘conditioned’ by social factors, its mere nature is relational since it could not exist outside of cultural resources and dialogical relations” (p. 88).

This relational perspective makes the notion of *affordances* relevant to the design of creativity enhancement interventions. Introduced by Gibson (1979/1986) as what environment “offers the animal, what it provides or furnishes, either for good or for ill” (p.127), affordances dictate how the surroundings guide, facilitate and constrain human activity. They are opportunities for action that emerge at the intersection between an actor’s idiosyncratic perception and the specific environmental ‘features’ (Chemero, 2003). In fact, Gibson (1979/1986) argued that action possibilities are the primary objects of perception and although affordances are not the cause of behavior, they have the potential to *invite* behaviors.

It is this concept of ‘*behavioral invitation*’ that matters for creativity intervention. Because affordances can both repel and attract actions, they play an important role in creative achievements (Glăveanu, 2013), even at the little-c level (Glăveanu and Kaufman, 2019). For instance, objects and spaces “channel” our actions by dictating what we can do with available instruments as well as where and how we can move within various spaces. Environmental manipulation thus impacts on what behavior is performed in a certain setting (e.g., Prieske et al., 2015). Yet, affordances are more than mere opportunity provided by the material world. Rietveld and Kiverstein (2014) suggest that affordances “have an existence that is relative to a form of life” (p.335). That is, the experiential knowledge we have as humans, of what objects and spaces are for, is influenced by cultural norms and social interactions. In this vein, because of the sociocultural practice we engage in, affordances are also “dependent on the abilities available in a particular ecological niche” (Rietveld and Kiverstein, 2014, p.326). This means that an affordance can be perceived (or not) differently by individuals coming from different sociocultural backgrounds and possessing a distinct set of skills and thus *invite* distinct behaviors which influence the likelihood of creative outputs.

To better exploit the relational and resourceful nature of affordances in the design of creativity intervention, the next section highlights the bidirectional transaction between actors and their cultural, social, and material context. We explore how the environment can itself be a source of creativity, one which, on one hand, influences the actor directly, and, on the other hand, can be influenced by the actor through its actions making “the whole an emergent property of the interactions of the members of a group or, more generally, the interacting parts of a system.” (Montuori, 2011, p.418).

#### Culture

“Culture is neither external to the person nor static, but constitutive of the mind and of society by offering the symbolic resources required to perceive, think, remember, imagine, and, ultimately, create” (Glăveanu et al., 2020, p.742). Creative thoughts and actions emerge from cultural knowledge and traditions – including one’s intention to disrupt traditions (Feldman, 1974). Culture can thus influence one’s perception and the expression of creativity. Consider for example the oversimplified conceptualization of collectivist versus individualistic cultures and the role of the latter over the former in creative-product outcomes (see Runco, 2014, for a review).

Beyond this, often-stereotyped, impact of culture on creativity at the societal level (i.e., macro-culture), cultural layers (e.g., homes, activities, schools, work places, institutions) have been hypothesized to intertwine with individual’s thoughts and behaviors through reward and tolerance systems (Runco, 2014). These systems, often implicit, depict the symbolic values accepted and/or rejected by individuals within a group impacting greatly how creative potential can be nurtured within each cultural layer (i.e., micro-cultures). Broadly, if creative ideas and behaviors are constantly reinforced by members of one’s own culture, then thinking and acting creatively slowly becomes a micro-cultural value increasing the likelihood of overt creative development and expression (Sternberg, 2006; Vaughan et al., 2019). More precisely, research have shown that cultural values oriented toward freedom, autonomy, risk taking, and playfulness are conducive of creativity enhancement in various domains (Hennessey and Amabile, 2010; Davies et al., 2013; Richardson and Mishra, 2018; Stierand et al., 2019). Yet, to translate into optimal environments, these values must be carefully instilled and accepted by people within each micro-culture (e.g., a sport team or a classroom).

For example, a systematic review of 210 educational research articles showed that creativity is enhanced when students are given some control over their learning and are encouraged to take risks within an environment that provides a balance between structure and freedom (Davies et al., 2013). In this vein, individual’s dispositions (e.g., level of self-control) and task conditions (e.g., prior experience with the task) was found to alter the function of task-autonomy in relation to creativity (Jin Wook et al., 2012). Therefore, to enhance creative potential “the degree to which an individual is given freedom and discretion in carrying out a task” (i.e., task-autonomy, Breaugh, 1985) should be balanced and contextualized. A similar tension must be maintained for risk-permissive cultural norms to lead to creative behaviors. That is, presenting and supporting enough risk to allow individuals’ to explore and embrace the unknown from which they can draw novel ways of thinking and acting, while providing boundaries for one to feel a sense of security (Creely et al., 2020). Finally, reasonable evidence supports the role of playful environments, at all ages, for the development of creative skills (e.g., Davies et al., 2013; Stierand et al., 2019).

The culture at the esteemed Cloud Gate Dance School in Taiwan described by David Mead (2009) as a “creative ethos” summarizes the equilibrium between frequently dichotomized elements constituting a creativity supportive culture: “Creativity at the school is not focused on external products but is more about process, innovation, and control. It is a creativity that incorporates a balance between control and freedom, collectivism and individualism, tradition and embrace of the new, and constraint and innovation” (p. 282). Although we are certainly products of the various macro and micro cultures we daily navigate (Runco, 2014), cultures are produced and reproduced. Therefore, how creative values are developed, shared, transmitted, and transformed through social interactions is a question that deserves further attention.

#### Social Interactions

If culture is about co-produced values and practices, social interactions are exchanges between individuals where these implicit structures are negotiated, reproduced and/or optimized into novel values that better shape social identity and experiences. Social interactions represent a crucial element of the creative system because actors are ‘socialized selves’ that navigate and influence sociocultural contexts through their cooperation with others (Hennessey and Amabile, 2010; Glăveanu, 2013). In Glăveanu’s (2013) terms, others are “audiences” which refer to any form of social support or pressure that comes from someone assisting, contributing, judging, criticizing, or using the creative act and/or resulting artifact(s). The audience role in a creative journey can thus be played by a variety of ‘others’ from collaborators, peers, family members, friends, opponents, teachers, critics, general public, and so on (Glăveanu and Kaufman, 2019). The actor and the audience are in constant dialog and their respective role are often dynamic meaning that it can change over time and among each other (Glăveanu, 2013). So, what actor-audience interactions are favorable for everyday creativity to happen?

Cultural values and practices encouraging freedom, autonomy, risk taking, and playfulness entail variable challenges for everyone actively part of that creativity supportive culture. For someone to perceive and positively navigate these challenges, the quality of the social interaction matters greatly. According to Edmondson and Mogelof (2006), the role of interpersonal relationships throughout the creative development process is crucial to help actors cope with perceived risk of failure, novelty, and ambiguity. Although risk-taking is promoted within a group, some individuals might need extra support from their leaders or peers to deal with their initial fear of making mistakes, losing their landmark, or feeling uncomfortable. Specifically, interpersonal climate, characterized by psychological safety, has been shown to lead to increased creativity in a number of different contexts including the workplace (Edmondson, 2002; Edmondson and Mogelof, 2006), the classroom (Davies et al., 2013), and the performing arts (Watson et al., 2012). Psychological safety describes interactional spaces that are safe for interpersonal risk taking and where speaking up about concerns, reporting mistakes, exposing thoughts, or suggesting new ideas is positively received. It stems from mutual respect and trust among group members providing to all, the confidence that they will not be ridiculed, rejected, or reprimanded for expressing different thoughts or behaviors (Edmondson, 1999; Edmondson and Mogelof, 2006).

Interpersonal trust, a core element of psychological safety, is of particular interest when it comes to enhancing creativity. According to Carmeli and Spreitzer (2009), because trust manifests one’s degree of vulnerability to another, it cultivates an open space where people can both share and generate new ideas which depicts connectivity (i.e., open and generative relationships). These authors showed that trust was indeed linked to innovative work behaviors through connectivity and thriving (i.e., experience of learning and vitality). Amongst other behaviors, frequent verbal interactions showing support, encouragement or appreciation within team settings was associated to increased connectivity hence broadening possibilities for actions and creativity (Losada and Heaphy, 2004).

In addition to relationships with peers, whether individuals feel safe to express their creativity has frequently been associated with leadership behaviors (Edmondson and Lei, 2014). For instance, inclusive leadership manifested by openness and availability, was positively related to psychological safety leading to increased involvement in creative work (Carmeli et al., 2010). Similarly, creative growth was made possible in newcomers when trust in leadership was rated as high in the workplace (Harris et al., 2013). In the classroom, teachers instilling trust by showing respect, care and tolerance for differences (Richardson and Mishra, 2018) as well as responsiveness (i.e., warmth or support; Zhang et al., 2020), were found to facilitate creative behaviors in students.

As underlined by Glăveanu (2013), creative actions emerge out of multiple reciprocal actor-audience interactions. Therefore, both parties must invest effort to establish trusting, connected and vulnerable relationships. Watson et al. (2012) observed many instances of collective relationship building effort “where young dancers and faculty either found or were in the process of finding the courage to embrace their fears and vulnerability regarding creativity. […] This reciprocal exchange is potentially of huge benefit to dancers, artists and teachers at whatever age and stage of their dance careers.” (p.166). Nurturing psychological safety should thus be seen as an important creativity igniter (Glăveanu, 2010a).

#### Material World

Because the material world is presented to us by others, interacted with in relation to others, and modified with the support of others, materiality is grounded within sociality (Corazza and Glăveanu, 2020). The material and physical spaces are thus important aspects of the environment that impact on creativity (Glăveanu, 2012). Nevertheless, the physicality of environments is often disregarded in creativity theories and research (Corazza and Glăveanu, 2020). As mentioned earlier, material objects are key to the emergence of creativity because they both constrain and allow creative acts to emerge (Glăveanu, 2013). In other words, “our imagination might be able to change something into anything, but even imagination is constrained by how objects are and what we know they are for” (Corazza and Glăveanu, 2020, p.5; refering to Vygotsky, 2004).

To circumvent the limiting impact of the cultural and social influences on the perception of materiality, some individuals have re-claimed public spaces and objects by using them in unorthodox manners. A salient example of this re-appropriation of public spaces is the emerging practice of Parkour; a form of free running that propose socially alternative physical cultures aimed at exploring how one’s body can move in communion with one’s urban physical environment. Sometimes referred to as urban gymnastics, “traceurs” (i.e., those who practice Parkour), balance on roof tops, slide on ramps, jump over cars, hang on fences, vault over benches, perform tumbling on sidewalks, etc. More than a mere acrobatic physical activity, Parkour is also a social critique that disrupts traditional “technocapitalist” views of urban physical and social landscape. By perceiving a city as a jungle gym and not a business jungle, traceurs’ “anarcho-environmentalist” resistance illustrates how using physical space differently opens a myriad of creative opportunities (see Atkinson, 2009, for extended discussion).

The bi-directional relation between traceurs’ mind-body and the environment they navigate brings back to light the notion of socio-cultural *affordances* introduced earlier. For instance, an experienced traceur may perceive a fence as an opportunity for testing a new vault to express agency whereas a businessman might perceive it as an obstacle that will make him late to his meeting. All opportunities are present for both individuals, yet the invitation to exploit the fence in a creative acrobatic way might only appear and be acted on by the traceur because it is supported by his/her system’s intrinsic dynamics (i.e., cultural values, cognitive skills, affective states, and physical abilities). The relationship between affordances, perceptions and action is an important mechanism underlying creative potential fulfilment. Exploiting the affordances of environments in a novel way, discovering new affordances, and even designing the ones needed to achieve a specific goal are considered creative actions (Glăveanu, 2013).

To summarize, “creativity emerges from playing with materials and acts of social relationality” (Stierand et al., 2019, p.166). On the one hand, creativity supportive cultural values make people more inclined to explore their environments, while social interactions make them feel safe to take risks, make mistakes and express their idiosyncrasies. Building on the literature reviewed, we suggest using the term *risk-friendly environments* to refer to socio-cultural contexts embedded in the material world that favor actors’ exploration of affordances. In line with the *tetradic cultural framework of creativity* (Glăveanu, 2010a), we thus advocate for a careful consideration of the environment as part of the design of activities aiming at developing embedded individual potential. Instead of focusing uniquely on developing creativity related skills in actors, practitioners and researchers should also assess the current state of an environment and adapt or manipulate certain elements to make it a risk-friendly one. Of course, to solicit or invite novel actions, the affordance of the environment must be complemented by actor’s effectivities (Rucinska and Aggerholm, 2019). Indeed, “Affordances make themselves ‘apparent’ only to an actor who is engaged with the environment and tries to navigate it effectively” (Glăveanu, 2012, p.194). The next section thus describes the actor’s elements that have been shown to facilitate the perception and usage of affordances in novel ways.

### Actor’s Elements

There are various attributes, skills, and states that dispose an actor to perceive and then act on affordances in ways that enhance his/her potential for creative growth. Accordingly, componential theories have been developed to gather the main personal attributes and abilities necessary for creativity to emerge (Kaufman and Glăveanu, 2019). For instance, the *Componential Model of Creativity* (Amabile, 1996; Amabile and Pratt, 2016) posits that *domain-relevant skills* (e.g., expertise, technical skills, and talent in a specific domain) *creativity-relevant processes* (e.g., tolerance to ambiguity and willingness to take appropriate risks) and *task motivation* are key inter-connected variables supporting individual creativity. Similarly, the *Investment Theory of Creativity* (Sternberg, 2012) holds that creativity requires a confluence of six distinct, but interrelated, resources grouped under three umbrellas; cognitive resources (i.e., thinking styles and knowledge), affective resources (i.e., motivation and personality), and environmental resources. Although we acknowledged that domain-relevant skills are essential for someone to reach pro-c and Big-C achievements (Baer, 2015), we adopt here a “system approach that operates at the level of generality necessary to address the contextual variation of creativity” (Poutanen, 2013, p.208). Consequently, the following focuses on creativity-relevant skills grouped under three interacting variables: affective attributes/states, cognitive skills, and physical expression. Following an embodied conceptualization of creativity, we consider these three variables as being closely (if not fully) intertwined. However, because creative potential is frequently associated with affective states and cognitive skills in the literature while the role of the body is ignored, we create distinct sections to review existing frameworks as a heuristic strategy, one that is not intended to imply there are separate elements. We aim at reuniting these variables in our suggested framework.

#### Affective Attributes and States

To navigate risk-friendly environments effectively, possessing or developing certain affective attributes and states can be helpful. In contemporary use, affect refers to the mental states that involve judgment and the conscious experience of feeling (Ekkekakis, 2012; Barrett and Russell, 2015). It encompasses a range of general, dispositional, time independent characteristics, to more time constrained concepts (Janelle et al., 2020). Early in the history of creativity research, scholars were interested in the association between dispositional characteristics and creative potential (Choi et al., 2020). For instance, results of a meta-analysis revealed that creative artists and scientists tend to be open to new experiences, self-accepting, hostile, and impulsive (Feist, 1998). More recently, building on the Big Two model of personality (Digman, 1997), *plasticity* – i.e., higher order factor encompassing openness to experience and extraversion – was found to be more strongly associated with creativity than *stability* – i.e., higher order factor encompassing neuroticism, agreeableness, and conscientiousness (see Feist, 2019, for a review). Specifically, “the strongest and most robust relationship between personality and creativity is the openness to experience dimension” (Feist, 2019, p.32). Because open-minded individuals are more inclined to explore, adapt to novel situations, question social norms, and seek out stimulating experiences, this disposition might facilitate the usage of affordances in creative ways.

Yet, the openness-creativity relationship is not strictly direct. Indeed, intrinsic motivation was found to play a mediating role in this relationship in undergraduate management students (Prabhu et al., 2008). Both componential theories position intrinsic motivation, defined as “the inherent tendency to seek out novelty and challenges, to extend and exercise one’s capacities, to explore, and to learn” (Ryan and Deci, 2000, p.70), to be one of the building blocks of creativity. In other words, it requires a strong drive and will to bring up new ideas or solutions in most socio-cultural contexts. In this vein, it was shown that the relationship between intrinsic motivation and creativity was mediated by the willingness to take risk in research and development employees (Dewett, 2007). Accordingly, the *Triangular Theory of Creativity* (Sternberg, 2018) stipulates that optimal levels of creativity result from a willingness to defy the crowd (e.g., people with more conventional beliefs), defy oneself (i.e., one’s own initial beliefs), and defy the Zeitgeist (i.e., spirit of time). This willingness to defy the *status quo* also comes hand in hand with the capacity of an individual to tolerate ambiguity. Indeed, “the tendency to perceive ambiguous situations as desirable” (Budner, 1962, p.29) is an attribute of creative individuals (Sternberg, 2018) which is especially useful when one encounters novel, complex and insoluble environments (Budner, 1962).

Risk-friendly environments may also elicit a myriad of moods and emotions thereby altering an actor’s level of openness, intrinsic motivation, willingness to take risk or tolerance to ambiguity. For instance, pleasant and unpleasant activating moods were found to influence employees’ level of tolerance to ambiguity which, in turn, altered their capacity to either find or solve problems creatively (Hwang and Choi, 2020). Moods and emotions are core affects defined as “neurophysiological state consciously accessible as the simplest raw (non-reflective) feelings” (Russell, 2003, p.148). The mood-creativity relationships has been extensively studied because mood often serves as a mediating state between situational and personality predictors and creative performance (Baas et al., 2008). According to the circumplex model of affect (Russell, 1980), mood can be classified based on two dimensions: activation and valence. Activation refers to the level of energy sensed while valence underlies the level of pleasantness experienced (Russell, 2003). The impact of the four mood categories (i.e., pleasant activating mood, pleasant deactivating mood, unpleasant activating mood, and unpleasant deactivating mood) on creativity can be partially explained through their impact on cognitive processes. Pleasant activating moods tend to broaden one’s scope of attention and promote cognitive flexibility whereas unpleasant activating moods increase the amount of ideas through their effect on persistence (De Dreu et al., 2008). The impact of both pleasant and unpleasant moods on creativity can also be facilitated by supportive social interactions (George and Zhou, 2007). On the other hand, because both pleasant and unpleasant deactivating moods such as calm and sadness lead to inaction and disengagement with the environment, they are less conducive to creativity (Baas et al., 2008). The impact of cognitive processes on creativity are explained more in depth in the following section.

#### Cognitive Skills

Among the cognitive skills associated with creativity, divergent thinking (DT) is a reliable and reasonably valid predictor of creative potential (Runco and Acar, 2012) and is the central construct behind most creativity tests (e.g., TTCT; Torrance, 1966). According to Guilford’s Structure of Intellect model (SOI; 1968), DT is the cognitive process associated with the generation of many alternative ideas. Specifically, facing an open-ended problem, DT encompasses the capacity of an individual to generate many ideas or solutions (i.e., *fluency*) that pertains to different categories (i.e., *flexibility*) and are unique contrasted with a sample dependent norm (i.e., *originality*).

The Dual Pathway to Creative Performance (DPCP; Nijstad et al., 2011) provides a more profound exploration of the cognitive processes underlying creativity. According to this framework, *persistent* and *flexible* thinking represents two functional ways to generate creative solutions. Specifically, to come up with novel associations, one must show persistence to focus and explore in depth a limited number of potential solutions (De Dreu et al., 2012). The fact that unpleasant moods such as anxiety signal a problematic situation, and thus force people to deploy additional effort to solve the challenge at hand, supports the positive link between unpleasant mood and creative solutions through persistence (George and Zhou, 2002). On the other hand, the same level of creativity might be achieved through flexible mind wandering. That is, defocused attention and latent inhibition allow many concepts coming from different sources within one’s attentional stream, which promote spontaneous creative insights. In this instance, a pleasant mood might signal a problem free situation facilitating effortless thinking and openness to ideas coming from diverse areas (Fredrickson, 2001).

Perspective taking has also been shown to be an important cognitive skill to unlock team creativity (Grant and Berry, 2011; Hoever et al., 2012). Importantly, a distinction must be acknowledged between divergent thinking theories and perspectival approaches to creativity. As described above, the former assume that ideas come to mind through pre-existing knowledge association. However, the latter considers the origin and dynamic of ideas as fundamentally social. The moment of insight is thus more than the result of cognitive processes; ideas are influenced by the perspective or action of a person in the world (Glăveanu, 2015).

Accordingly, generating fluid, flexible, and original thoughts is not sufficient to become creative. One must possess or develop the confidence to take action on those thoughts and share them with others. This transition from creative potential to creative accomplishment at the everyday level represents an agentic action depicting a person’s choice (conscious or not) to think and act in a novel way. The *Creative Behavior as Agentic Action* (CBAA) posits that this decision to act creatively is influenced by two keys factors: creative confidence and perceived value of creativity (Karwowski and Beghetto, 2019). Creative confidence encompasses two types of self-beliefs. On one hand, *creative self-efficacy* (CSE) is a dynamic and prospective belief in one’s ability to perform creatively a given task, in a specific context, at a particular level. On the other hand, *creative self-concept* (CSC) refers to a more holistic cognitive and affective judgment of one’s creative ability in and across particular domains (Beghetto and Karwowski, 2017). Finally, perceiving creative activities as worthwhile is essential. In a series of studies, Karwowski and Beghetto (2019) confirmed that creative potential works through creative confidence to influence creative behavior and that valuing creativity moderate both the creative potential-behavior relationship and the creative confidence-behavior one.

Although the CBAA framework acknowledge the essential connection between creative thoughts and actions for someone to realize his/her creative potential, the role of the body in this relationship is still unclear. In fact, the role of physical expression has been either ignored or only indirectly addressed by most actor-oriented frameworks presented above underlying their dualistic tendencies which prevent them to address the complex interactions linking the mind-body-environment. To adopt an embodied perspective, a deeper dive into the physicality of creativity is needed.

#### Physical Expression

In synergy with affects and cognitions, the body participates in the emergence of creative actions. Accordingly, a recent framework developed by Creely et al. (2020) conceptualized creativity as a way of being, expressing, emerging, and existing. The first mode of their *Three Modes of Creativity* model considers creativity as a “visceral embodied expression in the physical world or as a set of tangible practices that are physically located in space and time” (p. 2). Embodiment is based on the premise that the brain and body are intrinsically coupled (Gallagher, 2015). When considered as a corporeal connection with others and the material world, embodied creativity often relates to domains such as music, painting, sculpture, and performing arts where senses, feelings, and the totality of the environment of things are engaged through the body (Creely et al., 2020).

In this vein, over the last decades, researchers from various branches of movement sciences have been exploring the body as a vehicle to express creativity. Specifically, motor creativity has been defined as the type of creativity based on movement, “in which the process of creativity is embodied and the body is part of the creative product” (Torrents et al., 2020, p.1). It can support the resolution of pre-established problems or the expression of an idea or an emotion by the means of the human body (Wyrick, 1968; Bournelli et al., 2009). Motor creativity has thus been linked to various types of creative artifacts from the emergence of novel dance movements (Torrents et al., 2015) to Olympics gold medal winning performance outbreak such as the “Fosbury flop” jump or the “Tsukahara vault” (Bar-Eli et al., 2008). Although motor creativity research is contributing to deepening the understanding of creativity as an embodied concept, the fact that creative movement and performance have mainly been considered as end points leaves underexplored the potential of moving creatively as a means for people to reach their full creative potential (Rasmussen et al., 2017).

### A Systemic Approach to Foster Creative Potential

The goal of the first section was to review the elements that have been linked to creative potential fulfilment. In accordance with the *embedded individual potential* definition (Corazza and Glăveanu, 2020), we depict the creative potential as a complex system where creative actions arise from highly interactive environmental and actor elements. For instance, when an actor’s openness, willingness to take risk, and cognitive flexibility, interact with a risk-friendly environment, the actor’s perception of affordances is facilitated, increasing the likelihood of creative actions to emerge. Hence, compared to unidimensional approaches adopted by many studies aiming at enhancing creativity (Scott et al., 2004; Valgeirsdottir and Onarheim, 2017), interventions should not only be about developing individual skills (e.g., divergent thinking) but also about improving environmental factors. To facilitate the emergence of creative actions, interventions should favor activities that integrate a combination of multiple elements of the system. We thus suggest that creative potential can be nurtured through a systemic approach because it captures interactions between various elements while providing different levels of explanation and perspectives which predict the emergence of creative actions (Poutanen, 2013).

**FIGURE 1:**
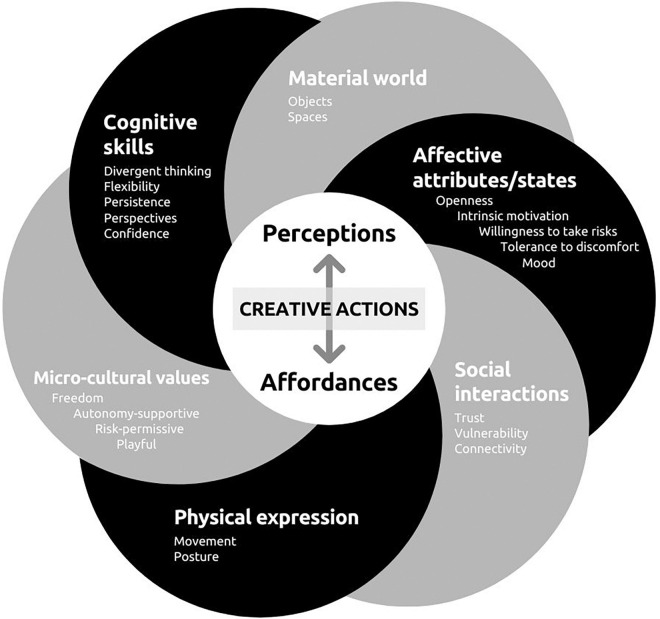
Interacting elements of the creative potential system.

If learning is defined as “the modification of a pre-existing repertoire that is unique to each individual” (Kelso, 2019, p.85), interventions must provide participants with opportunities to challenge their creative potential system repertoire at multiple levels. Building on the review, Figure 1 represents the elements of an individual system repertoire that could be supported and/or challenged to foster someone’s creative potential. It summarizes ‘what’ a creativity enhancement intervention should be directed toward. Whereas the review divided the elements in distinct sections, Figure 1 reunites them to emphasize a holistic and embodied perspective of creativity. The black strand represents the actor’s elements while the gray strand represents the environmental ones. Both strands are intertwined to support creative actions through these interactions influencing the perception of affordances. Of course, not all systems initially present facilitating interacting elements. Each element act as rate limiters on the emergence of creative actions (Renshaw et al., 2009). This can be exemplified by actors exhibiting cognitive rigidity or environments governed by an authoritarian culture limiting the creative potential of the system. In this latter case, well designed creativity enhancement interventions could potentially improve the system or provide additional resources to provoke or accelerate the growth of creative potential.

Because “perception, cognition, emotion, human relations, and behavior are grounded in our bodies” (Marmeleira and Duarte Santos, 2019, p.410), whether body-oriented creativity training could be a holistic way to enhance the creative potential system is a path worth exploring (Valgeirsdottir and Onarheim, 2017). As mentioned previously, the transformational potential of using creative movement to nurture the creative potential system has been underexplored. Hence, using philosophical, theoretical, and empirical movement science perspectives, the second section of this paper sheds light on the potential of movement-based activities to design creativity enhancement interventions.

## The Holistic Nature of Enriched Movement Activities and Its Impact on Creativity

From a philosophical standpoint, to move away from Cartesian dualism casting the body as a mere ‘home’ of human intellect, existentialist and phenomenological thinkers, such as Sartre and Merleau-Ponty, highlighted the pivotal role played by our embodiment in life. Both advocate for “the indispensable contribution made by our embodiment in, for example, self-realization, perception, concept development, language formulation, rationality, emotion and the development of interpersonal relationships” (Whitehead, 2007, p.283). Embodied activities thus have the potential to stimulate simultaneously many aspects of the creative potential system intrinsic dynamics. In other words, if “for human reality, to be is to act” (Sartre, 1957, p. 474), then becoming more creative should too be developed through actions.

Following this perspective, the few embodied creativity training programs that were empirically tested have used action-oriented activities (Byrge and Tang, 2015). For instance, role play training positively impacted creative imagination, thoughts and values in undergraduate students (Karwowski and Soszynski, 2008), creative drama increased participants’ fluent and flexible thinking (Karakelle, 2009), and an embodied creativity training program contributed to the enhancement of creative production, self-efficacy, and attitude (Byrge and Tang, 2015). While those interventions focused on forms of embodiment that mainly involve verbal and/or art expression to impact specific actor’s creative cognitive and affective skills, they did not directly challenge the body or integrate movement *per se*. We believe that there are additional benefits in adopting a motile embodied perspective to enhance creative potential.

In this vein, some studies tested the impact of various movement activities on creative potential. For instance, a 20-min aerobic dancing session significantly impacted DT scores in female college students (Gondola, 1987). Similarly, findings revealed that aerobic exercise (jogging, swimming, fast walking, stationary biking, or stair climbing), enhanced DT in physically fit students, both immediately and 2 h after completing the workout (Blanchette et al., 2005). Other evidence suggested that acute aerobic exercise benefits athletes’ creative performance through improved convergent thinking skills while impairing creative scores in non-athletes (Colzato et al., 2013). These findings are mainly explained in terms of the physiological impact of exercising on creative cognition, ignoring the wholeness of movement activities as well as all other element associated with creative potential. To circumvent these limitations, we explore how embodied activities can become enriched experiences.

### Physical Literacy

One way of thinking about embodied movement is through the lens of what scholars in physical education refer to as physical literacy (Dudley et al., 2017). We introduce it here given the rapid uptake of the concept by practitioners and influential non-governmental organizations such as the WHO and UNESCO. According to Whitehead (2007), “the capacity to capitalize fully on our embodied dimension could be encapsulated in the term physical literacy” (p. 286). Broadly, physical literacy can be defined as “the motivation, confidence, physical competence, knowledge and understanding to value and take responsibility for maintaining purposeful physical pursuits/activities throughout the life course” (Whitehead, 2013, p.29). Moving away from fundamental movement skills, a recent conceptual model describes physical literacy as the “multidimensional, experiential convergence of motor, affect, social and cognitive components” interacting with various physical environments (Cairney et al., 2019, p.373).

Activities recruiting physical literacy can thus be conceived as enriched multidimensional learning experiences (Roetert et al., 2017). Not only is moving essential for health and increasing movement proficiency, but it can also alter cognitive, affective, social, and cultural repertoires. For instance, a community-based movement skill and preliteracy program produced synergistic gains in both gross-motor and preliteracy skills in preschool-aged children in addition to facilitating the engagement of parents in such activities at home (Bedard et al., 2017). In a cross-sectional study conducted on children aged 9–12 years old, physical literacy was found to be a predictor of resilience. Specifically, authors concluded that “if the affective domains of confidence and motivation developed in physical literacy go beyond just motor action, then they may also provide or help young people acquire the skills and abilities to better negotiate for, and navigate to, resources that sustain their well-being in different contexts” (Jefferies et al., 2019, p.4). Although not specifically referring to physical literacy, a recent review of embodied-related literature highlighted the holistic benefits of various movement-based interventions such as dance/movement therapy to help people achieve new states of mind or body-exercises to support mood, emotions, and cognition in older adults (Marmeleira and Duarte Santos, 2019). These studies support the assumption that physical literacy provides insight into creating optimal physical-psycho-social environments that could foster the development of key processes underlying creativity (Jefferies et al., 2019). To achieve this goal though, some principles must be followed to design enriched movement experiences.

### Non-linear Pedagogy as a Guiding Principle to Design Enriched Movement Activities

Not all movement-based activities provide the same level of ‘enrichment.’ In movement sciences, the term enrichment refers to an embedded approach to motor learning which highlights rich and varied possibilities to achieve task goals through interactions constrained by the body, the task, and the environment (Rudd et al., 2020). For instance, rather than using repetitive prescriptive learning methods to acquire one single ‘optimized’ movement pattern, non-linear pedagogy (NLP) stipulates that, because there is an infinite number of individual intrinsic dynamics (Phillips et al., 2010), it is by manipulating specific constraints, removing barriers, and increasing freedom, that movers uncover novel, flexible, and functional movement patterns (Chow et al., 2006; Hristovski et al., 2006). Specifically, NLP is informed by *non-proportionality* (i.e., small changes in the system may significantly impact learners’ behaviors), *multi-stability* (i.e., one cause may have multiple behavioral effects), functional role of *noise* (i.e., variability in the system dynamics is encouraged), and *parametric control* (i.e., manipulating specific parameters to effectively guide a learning system) (Chow et al., 2011).

To design enriched movement experiences and inspire creativity, the manipulation of key constraints is central to NLP (Chow et al., 2006, 2011; Santos et al., 2016). Defined as “boundaries or features that shape the emergence of behavior by a learner seeking a stable state of organization” (Chow et al., 2006, p.262; refering to Newell, 1986) constraints are classified among three categories: organismic, environmental, and task related. Organismic constraints refer to personal characteristics that are either *structural* meaning they remain relatively stable over time (e.g., body composition and personality) or *functional* implying a faster change rate potential (e.g., fatigue, heart rate, motivation, and cognitive state). Environmental constraints encompass all those outside of the person such as climate, apparatus, material, and sociocultural factors. Task constraints can be considered as being *instructional* such as rules and instructions provided by a leader or *informational* such as visual or acoustic cues coming from the environment (Newell, 1986).

The same way affordances are relational, task constraints have recently been conceptualized as distributed between the environment and the organism. This means they are emergent properties of the organism-environment system. Because the material environment can only become a task constraint when interacting with a goal-oriented organism (‘actor’ in this paper), designing an enriched movement task to foster creative potential implies designing an inviting relation between the actor and the environment. Furthermore, organismic, environmental, and task constraints are nested in timescales impacting behaviors differently. Faster-changing constraints (e.g., perceptions, emotions, social interactions) have short-term effect on behavioral variables while slower-changing constraints (e.g., personality, cultural norms) have more durable impacts on behaviors. A circular causality relationship links these constraints as intervention at the slow-changing constraint level support changes at the fast-changing level and vice-versa (see Balagué et al., 2019, for more details).

Constraining the actor-environment system to stimulate creative movement may sound paradoxical since we argued earlier in the paper that creativity was linked to free, autonomous, and risk-permissive environments. Nevertheless, whether it is a painter that self-imposes a technique to explore something different or a circus artist that uses a piece of equipment to force his/her body toward new directions, the careful manipulation of constraints can lead to the exploration of underused repertoire potentially facilitating the discovery of novel solutions (Torrents et al., 2020). This assumption is supported by studies looking to improve movement creativity in various populations and settings. For example, constraining the distance to a punching bag target while asking novice boxers to perform efficient strikes led to the exploration of a rich range of striking actions (Hristovski et al., 2006). Similarly, the manipulation of hand hold, design to instill uncertainty and instability in climbers’ motor system, impacted positively on exploratory behaviors (Orth et al., 2018). In dance, Torrents et al. (2015) explored the effect of evolving constraints (free dancing, pelvis as close as possible, and pelvis as far as possible) on three pairs of contemporary dancers. Results indicate that task constraints have a significant effect on the type of configurations performed by the dancers. In a more ecological setting, a conventional elementary school fitness program was metamorphosed into a more creative one using NLP. The use of constraints, variability, fantasy play, and problem solving to encourage children to move differently resulted in significantly more original thoughts, as well as fluent and flexible movements compare to the children that continued the conventional exercise program (Richard et al., 2018).

In sum, these studies support the idea that perturbating the motor system by constraining ‘traditional’ or ‘familiar’ movement patterns while allowing the freedom to explore various solutions create enriched learning experiences in which creative movement are more inclined to emerge. What remains underexplored though is the impact of such enriched movement activities on the other elements of the creative potential system. As mentioned earlier, the research connecting movement and creativity focuses mostly on optimizing motor performance (Rasmussen et al., 2020). However, because of the holistic nature of movement underlined in this section, there are reasons to believe that enriched movement activities can also impact the system at the affective, cognitive, social, and cultural level. The next section introduces *movement improvisation* to exemplify how enriched movement activities can ignite the whole creative potential system.

## Movement Improvisation as a Creative System Igniter

Improvisation presents many assets to design effective learning experiences and challenge the creative potential system. Defined as the act of creating something new, on the spur of the moment, improvisation helps people break away from set patterns (Lewis and Lovatt, 2013). Improvisation is a complex form of creative behavior (Beaty, 2015) since individuals must process and act on several stimuli simultaneously (Torrents et al., 2020). Furthermore, improvisational activities are particularly well-suited for creative growth because (a) the process is the creative product, (b) it promotes respectful and attuned actors-environment interactions, (c) it is highly unpredictable and thus can perturbate the system (Sawyer and Dezutter, 2009; Malinin, 2019).

In line with NLP characteristics (Chow et al., 2011), when improvising in group setting, every small change in an action performed by one individual can impact another person’s action greatly (i.e., non-proportionality). Then, each improvisational task presents multiple response possibilities (i.e., multi-stability). Finally, while the unpredictable nature of improvisation creates a lot of ‘noise’ and variability in the system, its interactive nature with the environment (human interactions and material world) can serve as parametric control constraining people possible responses. In short, improvisation can provide a risk-friendly environment where improvisers must consider their personal constraints (i.e., cognitive skills, emotional state, physical competency, etc.) as well as the environmental ones (i.e., actions of other performers, space, material, surface, etc.) in order to navigate the task (Torrents et al., 2020).

The frequent use of improvisational technique has been associated to the development of creative assets in various domains. For instance, findings revealed that jazz musicians, who are highly skilled in improvising, have higher ideational creativity, are more open minded, and produce more creative musical achievement compared to classical musicians (Benedek et al., 2014). Similar results were found when comparing contemporary dancers (i.e., freely improvise on stage) to classical ballet dancers (Fink and Woschnjak, 2011). In this vein, contact improvisation was found to provide the right balance between collective constraints and individual freedom, which facilitated the emergence of creative movement in dancers (Kimmel et al., 2018). Other than for its performative purpose, improvisation was successfully used to improve undergraduate students’ fluency, flexibility, and originality scores (Lewis and Lovatt, 2013) as well as positive affect and uncertainty tolerance (Felsman et al., 2020). In line with the idea that improvisation can contribute to the fulfillment of creative potential elements (and not only achievements), the implementation of theatrical improvisation classes led to positive effect on self-concept in children (DeBettignies and Goldstein, 2020) whereas dance improvisation was associated to cognitive and socio-emotional growth in students by experienced dance teachers (Biasutti, 2013). Interestingly, improvisation techniques derived from jazz music were used to disrupt the dominant coach-centered culture rooted amongst sport coaches, to promote conversational exchanges (i.e., collaborative approach) between them and players (Santos and Morgan, 2019). The intervention impacted players’ capacity to generate creative solutions when facing challenges thereby highlighting the sociocultural impact of improvisation on creative potential. Studies implementing improvisation interventions to challenge the creative potential system as a whole remain scarce leaving us with a poor understanding of the depth of impacts these activities can have especially when combined with movement. Consequently, the focus here is to shed light on the potential of using improvisation with movement as a creative system igniter.

Inspired by *Cirque du Soleil* former talent development program, physical comic and theatrical improvisation techniques were employed to design a 20-h intervention aiming at helping elite figure skaters free up their performance and optimize their mental states. Not only quantitative measures of creative attitudes and values improved significantly, but skaters reported being more open-minded, willing to take risks, and flexible following the intervention. Additionally, skaters noticed how improvisational activities helped build relationships with other participants (Richard et al., 2017). After gathering scientific, empirical, and experiential evidence, an intervention called *movement improvisation* was designed (i.e., no use of verbal skills) to challenge the elements of the creative system. Movement improvisation is a series of activities conducted in a group setting that are regulated by two ‘rules’ to establish the micro-cultural values at the onset of each session. The first rule invites participants to “leave their judgments at the door; the judgment of others, but most of all the judgment of themselves.” The second rule stipulates that there are no other rules. The participants are thus allowed to respond to the stimuli suggested by the different improvisation activities in any way they want. These initial steps are taken to establish a risk-friendly environment.

At the start of each session, an interactive warm up activity is implemented to help participants connect with each other and get their body moving. This moment also allows the instructor to get a sense of both individual and group dynamics, which must be appropriately challenged throughout the session to nurture creative potential. Although movements are directly constrained by various stimuli, improvisation activities are meticulously planned to challenge usual ways of thinking, feeling, behaving, and interacting with others as well as the material world through movement. Movement improvisation task constraints thus emerge at the intersection between organismic constraint such as moving at random speed or while mimicking something or to express emotions and environmental constraints such as the use of different style of music and the establishment of various social contexts (i.e., individual, dyadic, and collective improvisation). In accordance with NLP, because there is no prescribed or expected ways to navigate each activity, participants are free to explore their boundaries, discover new ways of navigating situations and adapt at their own rhythm. Finally, each activity is followed by a debrief that encourages participants to reflect on their physical (e.g., what have you noticed in terms of movement and physical sensations?) cognitive (e.g., any specific thoughts during the activity?), affective (e.g., how did you feel when…?) and social (e.g., how did you navigate the situation together?) experience.

To exemplify the process, the following describes one of the basic activities titled “walking away from the obvious.” In this improvisation, participants are asked to walk in the room while imagining that their body is made of various substances (e.g., how would you walk if you were made of water, wood, oil, etc.). The body is thus ‘constrained’ to explore other ways of walking by embodying their interpretation of various substances (i.e., organismic constraint) while being primed by a slow and soft music (i.e., environmental constraint). Because the substances are randomly introduced, it also challenges participants’ flexibility of both movement and thoughts to transition and transform their way of walking from one substance to the other. Additionally, it solicitates their openness to reconsider the usual way to walk, willingness to take risk to push movement solutions in original directions, and vulnerability because everyone looks somewhat silly and feels a level of discomfort while embodying substances.

To start investigating the potential of movement improvisation as a creative potential enhancement intervention, a 5-week program was designed and compared to aerobic dancing and a control condition in a study testing the differentiating effects of these conditions on motor creativity and divergent thinking. Although findings revealed a significant effect for both movement improvisation and aerobic dancing on motor creativity variables compared to control, the effect sizes of movement improvisation were greater. Moreover, only movement improvisation impacted significantly original thinking compared to the control condition (Richard et al., 2020).

These preliminary results combined with the study using *Cirque du Soleil* intervention partially support the assumption that enriched movement activities (e.g., movement improvisation) can support the growth of multiple elements of the creative potential system, simultaneously. In other words, enriched movement activities do not only lead to creative movement, they can also enhance cognitive, affective, social and cultural elements associated with creative potential. To help researchers and practitioners to design more enriched movement activities aimed at enhancing the creative potential system, understanding the mechanisms of change is key. Yet, how creativity enhancement intervention works is still underexamined in most mainstream creativity research (Valgeirsdottir and Onarheim, 2017). To bridge this last gap, the next section describes the mechanisms explaining how enriched movement activities can optimize the creative potential system.

## How Does Enriched Movement Activities Provoke Changes in Creative Potential System?

“Complex systems are those with very many independent degrees of freedom (roughly component parts)” (Davids et al., 1994, p.501). Due to the complex and dynamic interactions underpinning the emergence of creative actions, as depicted in the first section, the creative potential system mechanism of change can be conceptualized using complexity theory (Kozbelt et al., 2010). Central to this theory is the notion that, when perturbated, complex systems as a whole self-organize and adapt to the environment to facilitate the emergence of new order (Poutanen, 2013). The impact of constraints on creative movement has been conceptualized and operationalized within the ecological dynamics theoretical framework; “an approach using concepts and tools of dynamical systems to understand phenomena that occur at an ecological scale” (Araujo et al., 2006, p. 656). Because NLP is building on this approach, this section first reviews the mechanism of change leading to the emergence of creative movements. However, the goal of this section is to explore how principles of ecological dynamics could also explain the changes provoked by enriched movement activities on the cognitive, affective, social, and cultural elements of the creative potential system.

### Creative Movements Explained Through an Ecological Dynamics Lens

The complexity and non-linearity of the creative potential system can be described by its *multistability*, meaning that it is composed of multiple stable states or what is also sometimes called *attractors*. These pre-existing states/attractors (i.e., intrinsic dynamics landscape) are shaped by prior experiences and dictates the very nature of learning and change. When perturbated (e.g., through constraints), multistability allows the system to switch rapidly among states/attractors to sustain functionality and meet environmental and/or internal demands (Kelso, 2012). Another way for the system to preserve functionality when one part of the system is perturbated is through synergies; “context-sensitive functional groupings of elements that are temporarily assembled to act as a single coherent unit” (Kelso, 2012, p.907).

Because learning is about acquiring new patterns of behaviors, stable states/attractors must be perturbated to allow the exploration of new states (Kelso, 2019). Once the system is perturbated by constraints, it falls into a state of disequilibrium (Davids et al., 1994; Vaughan et al., 2019). The instability caused by internal and/or external constraints on the system triggers self-organization mechanisms which refers to “the spontaneous formation of pattern and pattern change in complex systems whose elements adapt to the very patterns of behavior they create” (Kelso, 2001, p.13845). Transition routes then become possible depending on the system initial intrinsic dynamics. On one hand, the *bifurcation route* reorganizes the system by adding new stable patterns implying novelty while the *shift route* generates smooth adaptation by altering the disposition of pre-existing patterns (Kelso, 2012). These two routes provide the minimum conditions for movement creativity to emerge (Hristovski et al., 2011). On the other hand, constraining the system can direct individuals toward metastable performance regions (Hristovski et al., 2006; Pinder et al., 2012; Orth et al., 2018) “where a system ‘hovers’ in a state of dynamic stability, switching between functional states of organization in response to changing constraints, and displaying subsequent behavioral flexibility” (Headrick et al., 2015, p.84). Because the system never really ‘settles’ in one state, metastability allows a flexible exploration and exploitation of affordances potentially resulting in novel and original movements (Chow et al., 2011; Hristovski et al., 2011). Because a person’s perception of affordances might be altered momentarily by these mechanisms, actions might also become unstable; they might get worse before they get creative.

The same way self-organization processes have been shown to lead to enhance creative movements (e.g., Hristovski et al., 2006; Orth et al., 2018, as described above), we suggest that it can explain how enriched movement activities can expand cognitive, affective, social, and cultural repertoire increasing the likelihood of creative actions emergence. Using movement improvisation, this assumption is exemplified next.

### The Mechanisms of Change Underlying Movement Improvisation

**FIGURE 2:**
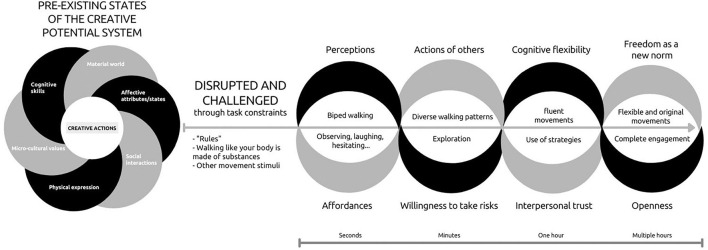
An example of a movement improvisation session mechanisms of change. The left intertwined strands image (i.e., summary of Figure 1) represents the pre-existing state of the creative potential system. Once the system is disrupted and challenged by a movement improvisation activity, the right part of the Figure presents the untied individual and environmental strands. We untied the strands to illustrate how the individual elements (black strand) and environmental elements (gray strand), while still interacting, hypothetically change on a time scale from a few seconds to a few hours. The mid-line arrow illustrates the direction of the chronology of these changes. Also, the white space above the mid-line arrow shows the emerging observable motor behaviors while the white space below the arrow presents other behaviors expressed throughout the activity.

So, how can we examine movement improvisation processes using ecological dynamics principles? Figure 2 illustrates the hypothetical mechanisms initiated during the ‘walking away from the obvious’ and operating throughout a movement improvisation session. Disrupted by the task constraints imposed upon established walking patterns (i.e., walking like substances) and the rules of the activity, the pre-existing state of each individual’s creative potential system is momentarily destabilized. Building on the idea that constraints are nested in levels and timescales (Balagué et al., 2019), Figure 2 exemplified changes for both actor (black strand) and environmental (gray strand) constraints and the resulting self-organized behaviors emerging from their interactions (i.e., The white space between the strands. Above the arrow line are the changes in motor behaviors while below the arrow line are the changes in other expressed behaviors). In the first few seconds of the ‘walking away from the obvious’ activity, participants’ perceptions of the affordances rapidly change. At this stage, a slight disequilibrium can be perceived by participants observing others, laughing, and hesitating. Participants are usually stuck within the attractor of ‘biped walking.’ Although the ‘rule’ of freely responding to stimuli in any way they want is well established at the onset, participants usually only perform movements that are close to the ‘biped walking’ attractor by embodying the substance with their arms or legs. They are mainly showcasing the *multistability* of the system alternating between stable states resulting in few original walking patterns.

Because the activity is conducted in a group, after a few minutes, one member of the group will usually ‘push’ the exploration to another level by starting to roll, crawl, or quadruped walk on the floor (‘walking,’ after all, is not limited to human bipedal motion only). Due to a change in the level of willingness to take risk, this participant moves away from the ‘biped walking’ attractor and starts, therefore, to explore unstable states. By doing so, it creates a competition between the pre-existing repertoire of the members of the group and influence the new behaviors to be achieved (Torrents et al., 2021). In other words, this changing environmental constraint ‘unlocks’ some participants from their main attractors and induces a metastable dynamic where participants can easily navigate between stable and unstable movements.

During a movement improvisation session, there is no motor performance expectations. The goal of improvising with movements is to perturbate the entire system to instill growth at all levels. So, what other cognitive, affective, social, and cultural changes happened to the system throughout a whole 2-h session? When debriefing with participants after the session, they often share how cognitively, affectively, and socially challenged they were. Some report not ‘knowing’ how to respond to certain stimuli (i.e., cognitively destabilize), having experienced anxiety or confusion (i.e., affectively destabilize), or being worried to look like a fool (i.e., socially destabilize). When we discuss the strategies they used to manage those experiences, some disclose focusing on the music, connecting with the body, entering a ‘bubble,’ imagining the stimuli, etc. As illustrated in Figure 2, after more than an hour, synergies form between participants increasing trust which led to the adoption of freedom and permission to take risk as shared values within the group. Changes in these environmental constraints can impact, for instance, on participants’ cognitive flexibility and openness which can be observed through enhanced fluency in movements. By the end of the session, these changes in constraints allow participants to completely engage and produce flexible and original movements. Because constraints correlated through circular causality, it is also important to note that adopting freedom as a group norm also impact the interpersonal trust which influence the actions of the participants in the group, making affordances more inviting. The same principle could apply for individual constraints. This supports the idea that affect, cognition, and behaviors as well as social and cultural factors also exhibit self-organizational tendencies which can transform the system intrinsic dynamics (Davids et al., 2001).

In summary, the movement improvisation constraints challenge more than movement patterns, it destabilizes the whole creative potential system. To regain stability, participants must explore multiple cognitive, affective, social, or cultural solutions to reorganize in more functional states. “Adopting novel and potentially functional states of system organization is a consequence of learning and/or development, as individuals transit from the ‘known’ to the ‘unknown,’ i.e., moving from a familiar task or situation to one that is new or different” (Headrick et al., 2015, p.84). This supports the underexplored idea that enriched movement activities can challenge the actor-environment creative potential system resulting in more creative actions, at least at the everyday level. Through movements, participants experience how they feel, think, behave, and interact when anchored patterns, such as walking, are disrupted. This embodied metaphor might facilitate the transfer of creativity-related skills to other activities (Malinin, 2019). It also broadens the envelop of creativity enhancement interventions by providing a rationale for movement activities as a holistic method.

## Challenges and Future Directions

### Challenges and Future Directions

Building on creativity and movement sciences literature, this review aims at highlighting the holistic power of movement as a creative potential system igniter. We argue that by directly perturbating the elements of the system through well-designed activities such as movement improvisation, cognitive, affective, social, and cultural repertoires can be enhanced. To move away from dualistic cognitive science perspectives and combine creativity and movement sciences, we build on ecological dynamics principles and an underlying pedagogical approach (i.e., NLP) to explain *how* and *why* enriched movement activities are promising candidates to provide the environment needed to support creative potential enhancement. To test the hypothesis raised in this paper and empirically unify movement and creativity sciences within an evidenced-based intervention, some challenges must be addressed.

The first challenge concerns the lack of knowledge about the impact of enriched movement activities on variables beyond motor variables. Studies using NLP or improvisation provide evidence for the benefit of tasks emerging from the manipulation of environmental constraints (e.g., distance, material, space) and/or organismic constraints (e.g., usual movement patterns) forcing the exploration of movement solutions resulting in the emergence of creative motor patterns (Torrents et al., 2020). However, to date, most movement science research has failed to address the nested organization in levels and timescales at the systemic level (actor’s and environmental elements) and their circular causality. Noteworthy, interventions triggering movement exploration rarely address the initial state of slow-changing elements such as personality (e.g., openness) and cultural values (e.g., risk-permissive) and their impact on faster-changing elements like motivation and cognitive states (e.g., flexibility). For instance, how is moving creatively influenced by a risk-permissive cultural value and influence cognitive flexibility and willingness to take risk?

To answer this question, researchers must shift away from “the empiricist preoccupation of reducing the universe to a series of simple, testable relationships” (Davids et al., 1994, p.501). Creative potential is defined throughout this article as a complex system where cultural values, social interactions, material world, and individual’s cognitive skills, affective states and physical expression are intricately linked (see Figure 1). Therefore, studying each of these elements individually “often disrupts their usual interactions so much that an isolated unit may behave quite differently from the way it would behave in its normal context” (Clarke and Crossland, 1985, p.16). The systemic and complex characteristic of creativity raises the thorny challenge of developing research and applied approaches that integrate rather than isolate interdependencies between all elements (Vaughan et al., 2019). According to Glăveanu et al. (2020), “even if one single study or intervention cannot address all these [creativity] dimensions simultaneously, the questions, methods, general design, and interpretation of findings should be chosen in view of this complexity” (p.742).

Building on this recommendation, we thus invite movement creativity researchers to look beyond movement outcome variables and start questioning whether and how disrupting usual movement patterns through enriched movement activities triggers the emergence of individual and environmental repertoire. Designing research questions and hypothesis using the model presented in Figure 2 could be a good starting point for researchers to examine more precisely the nested organization of variables in levels and timescales and provide a much more complete picture of the impacts of enriched movement activities on creative potential elements.

Using this approach raises the challenge of measurement. To assess changes in the creative potential system, one must assess its intrinsic dynamics (Kelso, 2019). Although efforts were deployed to develop measurement taxonomies such as the *heuristic framework for creativity measurement* that provide a multidimensional measurement structure (see Batey, 2012, for a review), most studies still adopt a siloed measurement approach. For instance, intervention studies usually used either variation of divergent thinking tasks to assess the cognitive elements (e.g., Torrance and Ball, 1984; Runco and Acar, 2012), self-reported measures to gather data about affective elements such as openness to experience, risk-taking, and motivational orientations (see Kaufman, 2019), or consensual assessment to evaluate the creative ‘product’ (e.g., Amabile, 1996). Yet, to reliably assess creative potential changes and developments throughout an intervention, unidimensional measurement approach is no longer sufficient. Optimizing measurement methods is thus pressing (Barbot, 2019). We believe Figures 1, 2 can serve as a useful guide to develop multidimension assessment tool.

We also call for ecological research designs where enriched movement activities are integrated as part of the regular ‘curriculum’ rather than isolated. Naturally, this suggestion makes sport organizations, exercise facilities and schools intuitively appealing milieu to conduct this type of applied research. But what would be the impact of integrating enriched movement activities in less intuitively movement-related domains such as medicine, architecture, or engineering where creativity is also key? What if before medical case study classes or brainstorm meetings, professionals were invited to improvise with movement? How would this transform the material, social and cultural environment and invite more creative behaviors thereafter? Could moving creatively regularly impact embedded individual creative potential and push these fields further? Longitudinal research adopting multidimensional measurement methods could answer these questions and support the benefits of integrating movement activities in all sorts of milieu keeping in mind that the goal is to improve how “people relate to the world, to others, and to themselves, making them more flexible, more open to the new and, at least in principle, to differences in perspective” (Glăveanu et al., 2020, p.743).

This cross-context approach however raises yet another challenge; the transferability of general training to other indirect (non-linear) outcomes. Of course, to become a eminent creator in any domain, specific training is needed to develop the appropriate skill set to move a field forward (Sternberg, 2012). Yet, as it was clearly established, the goal of using movement activities is to foster creativity-related skills. So, how does transferability apply to ‘general’ creative skill development? Like certain movement competences (e.g., agility, endurance, flexibility, power and stability) can be developed through non-specific experiences and then transferred to a specific sport (Rudd et al., 2020), we argue that the foundational elements of the creative potential system can be improved through enriched movement experiences. Accordingly, “it is suggested that the role of behavioral enrichment, conferred by generality of transfer has typically been misunderstood and undervalued in athlete development programs” (Rudd et al., 2020, p.7). In attempt to circumvent this, researchers have been interested in the role of *Donor Sport* to enriched foundational skills development and enhance a whole array of skills transferable to sport performance (Strafford et al., 2018). For instance, Parkour (described above) has been shown to be a suitable donor sport because it can ‘donate’ agility (i.e., physical skills), problem solving, risk-management and self-efficacy (i.e., psychological skills) as well as initiative and receptiveness to feedback (i.e., social skills) (Strafford et al., 2020). Following this argument, we suggest that creative movement activities such as movement improvisation can be considered *donor activities*. Future research should thus test whether movement activities can ‘donate’ affective, cognitive, social, and cultural elements to enable people to fulfill their creative potential in any domain.

## Conclusion

Imagine if athletes’ warm up would be designed not only to get their body prepared, but also to ready their mind to be more creative during training sessions. Imagine if creative movement sessions would be offered in most local gyms where you can both improve your creative fitness for body and mind. And what about integrating movement improvisation sessions before your next innovation session at work? What would be the impact on creative performance, but most of all, on well being? There are “parallels in teaching for creativity and promoting well-being; that the same elements which nurture creativity also promote well-being more generally” (Watson et al., 2012, p.169) and we argue that enriched movement activities could amplify these parallels.

Itself a creative exercise, this review connects movement to existing theories of creativity to highlight which elements creative enhancement interventions should target, how enriched movement activities can be designed, and by which mechanisms it can nurture the creative potential system. Although much work remains to be done to support these hypotheses, we believe that fostering creative potential through movement is a path worth exploring. Because while our world is in constant motion forcing our mind to sprint to find new ideas, our bodies have never been in such a state of inertia.

## Author Contributions

VR, DH, and JC contributed to the conception and design of the manuscript and models. VR conducted the literature review and led the drafting of the manuscript. DH and JC did extensive reviews of each version of the manuscript adding significant contributions to the content of specific sections. All authors contributed to manuscript revision, read, and approved the submitted version.

## Conflict of Interest

The authors declare that the research was conducted in the absence of any commercial or financial relationships that could be construed as a potential conflict of interest.

## Publisher’s Note

All claims expressed in this article are solely those of the authors and do not necessarily represent those of their affiliated organizations, or those of the publisher, the editors and the reviewers. Any product that may be evaluated in this article, or claim that may be made by its manufacturer, is not guaranteed or endorsed by the publisher.
